# Supplementary material for: Research groups: How big should they be?
Source: PeerJ. 2015 Jun 9;3:e989. doi: 10.7717/peerj.989 (PMC4465944; doi:10.7717/peerj.989)
Supplement: Supplemental Information 2 — File contains three emails that were sent to principle investigators during the project. [file peerj-03-989-s002.docx]

**Email sent by Isabelle Cook (autumn 2012)**

Dear professor/Dr…

I am a third year Biology student at the University of Sussex and am currently undertaking my final year project alongside Professor Adam Eyre-Walker. In order to understand the research infrastructure in the United Kingdom, we are conducting an analysis of research group size within the biological sciences. I would be very grateful if you could provide me with the number of individuals in your research group, reporting separately the numbers of PhD students, post-doctoral researchers and technicians. Please count individuals as fractions of full-time equivalents if the individuals are either part-time of shared with another research group. Please return your information by replying to this email. All data will be treated as strictly confidential and will not be shared with anyone outside the research group except where it has been anonymised.

We thank you for your cooperation.

Yours sincerely

Isabelle Cook and Adam Eyre-Walker

School of Life Sciences

University of Sussex

Brighton

BN1 9QG

http://www.sussex.ac.uk/lifesci/eyre-walkerlab/

**Email sent by Sam Grange (autumn 2013)**

Dear Sir / Madam,

I am a final year BSc Biology undergraduate at the University of Sussex, and I am conducting a survey of research group sizes in UK-based Biology departments as part of my dissertation. All information will remain strictly confidential and will only be seen by those directly involved in this research. My project supervisor is Prof. Adam Eyre-Walker ([A.C.Eyre-Walker@sussex.ac.uk](mailto:A.C.Eyre-Walker@sussex.ac.uk)).

I would be grateful if you could assist me with this research by replying to this email with the following information:

i. Research group size - inlcuding all PhD, PostDoc, & technicians (If a members time is split between various projects, please provide proportion devoted to your to the nearest 25%).

ii. Whether these members were at the same institution 5 years prior.

iii. The gender of the individuals in your research group.

Thank you for your time,

Sam Grange

**Email sent by Adam Eyre-Walker (Summer 2014)**

Dear Colleague

A couple of years ago you were contacted by one of my research students, Isabelle Cook, who was studying various aspects of research group size. You kindly responded to her email. As part of our continuing analysis of this dataset we would like to know whether you were employed at the same institution between Jan 1st 2008 and Dec 31st 2012, or whether you moved during this period. By institution we mean the university or research institute at which you were employed during this time, not the department or school. If you could supply me with this information I would be very grateful. All information will be treated as confidential.

Yours sincerely

Adam Eyre-Walker

Professor of Biology

School of Life Sciences

University of Sussex

Brighton

BN1 9QG
